# Signal transduction pathways involved in proteolysis-inducing factor induced proteasome expression in murine myotubes

**DOI:** 10.1038/sj.bjc.6601328

**Published:** 2003-10-28

**Authors:** H J Smith, M J Tisdale

**Affiliations:** 1 Pharmaceutical Sciences Research Institute, Aston University, Birmingham B4 7ET, UK

**Keywords:** proteolysis-inducing factor (PIF), phospholipase A_2_ (PLA_2_), phospholipase C (PLC), tyrosine kinase, mitogen-activated protein kinase (MAPK), proteasome expression

## Abstract

The proteolysis-inducing factor (PIF) is produced by cachexia-inducing tumours and initiates protein catabolism in skeletal muscle. The potential signalling pathways linking the release of arachidonic acid (AA) from membrane phospholipids with increased expression of the ubiquitin–proteasome proteolytic pathway by PIF has been studied using C_2_C_12_ murine myotubes as a surrogate model of skeletal muscle. The induction of proteasome activity and protein degradation by PIF was blocked by quinacrine, a nonspecific phospholipase A_2_ (PLA_2_) inhibitor and trifluroacetyl AA, an inhibitor of cytosolic PLA_2_. PIF was shown to increase the expression of calcium-independent cytosolic PLA_2_, determined by Western blotting, at the same concentrations as those inducing maximal expression of 20S proteasome *α*-subunits and protein degradation. In addition, both U-73122, which inhibits agonist-induced phospholipase C (PLC) activation and D609, a specific inhibitor of phosphatidylcholine-specific PLC also inhibited PIF-induced proteasome activity. This suggests that both PLA_2_ and PLC are involved in the release of AA in response to PIF, and that this is important in the induction of proteasome expression. The two tyrosine kinase inhibitors genistein and tryphostin A23 also attenuated PIF-induced proteasome expression, implicating tyrosine kinase in this process. PIF induced phosphorylation of p44/42 mitogen-activated protein kinase (MAPK) at the same concentrations as that inducing proteasome expression, and the effect was blocked by PD98059, an inhibitor of MAPK kinase, as was also the induction of proteasome expression, suggesting a role for MAPK activation in PIF-induced proteasome expression.

Loss of muscle mass is a debilitating and life-threatening feature of cancer cachexia, as well as a number of other catabolic conditions such as sepsis, burn injury, metabolic acidosis, severe trauma and denervation atrophy. In all these conditions, muscle wasting is due to accelerated muscle protein breakdown, combined with decreased protein synthesis. The major proteolytic pathway considered to be responsible for the increased protein catabolism in skeletal muscle is the ubiquitin–proteasome proteolytic pathway (Lecker *et al*, 1999). In this process, proteins are marked for degradation by attachment of a polyubiquitin chain through a series of enzymes (E1, ubiquitin-activating enzyme; E2, ubiquitin-conjugating enzyme; E3, ubiquitin–protein ligase) and are hydrolysed to peptides within a large (2000 kDa) 26S proteasome in a process that is ATP dependent. An increased expression of proteasome subunits and E2_14k_ has been observed in the gastrocnemius muscle of cachectic mice (Lorite *et al*, 1998) and rats (Temparis *et al*, 1994), suggesting these elements to be important in the increased protein degradation, although recent studies (Bodine *et al*, 2001) suggest that E3 may be rate limiting in ubiquitin conjugation.

We have isolated and characterised a sulphated glycoprotein (Todorov *et al*, 1996a), which initiates catabolism of skeletal muscle proteins both *in vitro* (Smith *et al*, 1999) and *in vivo* (Lorite *et al*, 1998), and for this reason has been called the proteolysis-inducing factor (PIF). PIF is produced by the cachexia-inducing murine MAC16 tumour (Todorov *et al*, 1996a) as well as murine colon 26, clone 20 variant, which induces cachexia, but not by clone 5, which does not induce cachexia (Hussey *et al*, 2000). Proteolysis-inducing factor is produced by human carcinomas of various types (Cariuk *et al*, 1997) and has been correlated with a significantly greater total weight loss and rate of weight loss in patients with pancreatic carcinoma (Wigmore *et al*, 2000). The induction of protein catabolism by PIF was shown to be due to upregulation of the ubiquitin–proteasome pathway both *in vivo* and *in vitro* (Lorite *et al*, 2001), suggesting a direct effect of PIF on this pathway.

Initial studies showed that protein catabolism induced by PIF was associated with the release of arachidonic acid (AA) and the conversion to eicosanoid metabolites of which 15(*S*)-hydroxyeicosatetraenoic acid (15(*S*)-HETE) was considered to play a central role in protein degradation (Smith *et al*, 1999). However, the mechanism by which this occurred and the relationship with proteasome and E2_14k_ expression was not determined. The most likely mechanism would involve phospholipase A_2_ (PLA_2_) acting on phospholipids releasing AA and a lysophospholipid, or by phospholipase C (PLC) with the formation of a diacylglycerol (DAG), followed by DAG lipase forming AA. The products of the PLC reaction (Nakamura and Nishizuka, 1994) as well as AA and lipoxygenase metabolites (Fan *et al*, 1990) are signalling molecules, which activate the protein kinase C (PKC) family of serine/threonine kinases, which we have shown (unpublished results) to act as intracellular signals of PIF action on the proteasome. The current study investigates the role of PLA_2_ and PLC on PIF-induced proteasome expression as well as on potential substrates for PKC using C_2_C_12_ myotubes, which we have previously shown (Gomes-Marcondes *et al*, 2002) to be a good model for studying PIF action on the proteasome.

## MATERIALS AND METHODS

### Materials

Fetal calf serum (FCS), horse serum (HS) and Dulbecco's modified Eagle's medium (DMEM) were purchased from Life Technologies (Paisley, UK). Mouse monoclonal antibody to 20S proteasome subunits *α*1, 2, 3, 5, 6 and 7 (clone MCP 231) were purchased from Affiniti Research Products (Exeter, UK), and rabbit polyclonal antisera to the ubiquitin-conjugating enzyme (E2_14k_) was a gift from Dr Simon Wing, McGill University, Montreal, Canada. The antibody recognises both isoforms of E2_14k_ encoded by HHR6A and HHR6B (Rajapurohitam *et al*, 1999). The HHR6B gene encodes the isoform for which mRNA levels increase in atrophying muscles. The antibody detected E2_14k_ as an *M*_r_ 17 000 band. Mitogen-activated protein kinase (MAPK) proteins and their phosphorylated (active) forms were detected with anti-extracellular signal-regulated kinase (ERK)1 and 2 [pTpY^185/187^] nonphosphospecific and phosphospecific rabbit polyclonal antisera (Biosource International, Belgium). Rabbit polyclonal antisera to PLA_2_, Type V1 was purchased from Calbiochem, Nottingham, UK. Sheep anti-mouse and goat anti-rabbit antisera were purchased from Dako Ltd (Cambridge, UK), while the *β*-tubulin mouse monoclonal antibody was obtained from Calbiochem, Nottingham, UK. The following inhibitors were also purchased from Calbiochem: quinacrine dihydrochloride, trifluoroacetylarachidonic acid, U73122, D609, genistein, tryphostin A23 and PD 98059.

### Cell culture

C_2_C_12_ myoblasts were grown in DMEM supplemented with 10% FCS, glutamine and 1% penicillin–streptomycin in a humidified atmosphere of 10% CO_2_ in air at 37°C. Myotubes were formed by allowing confluent cultures to differentiate in DMEM containing 2% HS, with medium changes every 2 days.

### Purification of PIF

PIF was purified from solid MAC16 tumours excised from mice with a weight loss between 20 and 25%. Tumours were homogenised in 10 mM Tris-HCl, pH 8.0 containing 0.5 mM phenylmethylsulphonyl fluoride, 0.5 mM EGTA and 1 mM dithiothreitol at a concentration of 5 ml g^−1^ tumour. The supernatant obtained after ammonium sulphate (40% w v^−1^) was subjected to affinity chromatography using an anti-PIF monoclonal antibody coupled to a solid matrix as described (Todorov *et al*, 1996a). The immunogenic fractions were concentrated and used for further studies. The only contaminant was albumin (Todorov *et al*, 1996b) and the PIF was used without further purification.

### Measurement of proteasome activity

The ‘chymotrypsin-like’ enzyme activity of the proteasome was measured using the fluorogenic substrate suc-LLVY-aminomethyl coumarin (0.1 mM) essentially according to the method of Orino *et al* (1991). Myotubes were washed in ice-cold phosphate-buffered saline (PBS) and sonicated in 20 mM Tris-HCl, pH 7.5, 2 mM ATP, 5 mM MgCl_2_ and I mM dithiothreitol at 4°C. The supernatant formed by centrifugation at 15 000 r.p.m. for 10 min at 4°C was analysed for ‘chymotrypsin-like’ activity using a Microplate Spectrofluorimeter (SPECTR max, Molecular Devices, CA, USA). Results were calculated as activity *μ*g protein^−^1 min^−1^. Protein degradation in the presence of PIF was determined as previously described (Gomes-Marcondes *et al*, 2002).

### Western blot analysis

Samples of cytosolic protein (2.5–5 *μ*g) were resolved on 10% sodium dodecylsulphate–polyacrylamide gels (SDS–PAGE) and transferred onto Hybond™ ECL™ nitrocellulose membranes (Amersham, UK), which had been blocked with 5% Marvel in Tris-buffered saline, pH 7.5, at 4°C overnight. The primary antibodies were used at a dilution of 1 : 40 (*β*-tubulin); 1 : 100 (E2_14k_); 1 : 500 (anti-ERK1 and 2) or 1 : 1500 (anti-20S proteasome), while the secondary antibodies were used at a dilution of 1 : 2000. Incubation was carried out for 2 h at room temperature and development was by enhanced chemiluminescence (Amersham UK). Loading was quantitated either by *β*-tubulin or by a parallel gel, which was stained with Coomassie brilliant blue.

### Statistical analysis

Results are expressed as means±s.e.m. Differences were determined by one-way ANOVA followed by the Tukey–Kramer multiple comparison test.

## RESULTS

**Figure 1:**
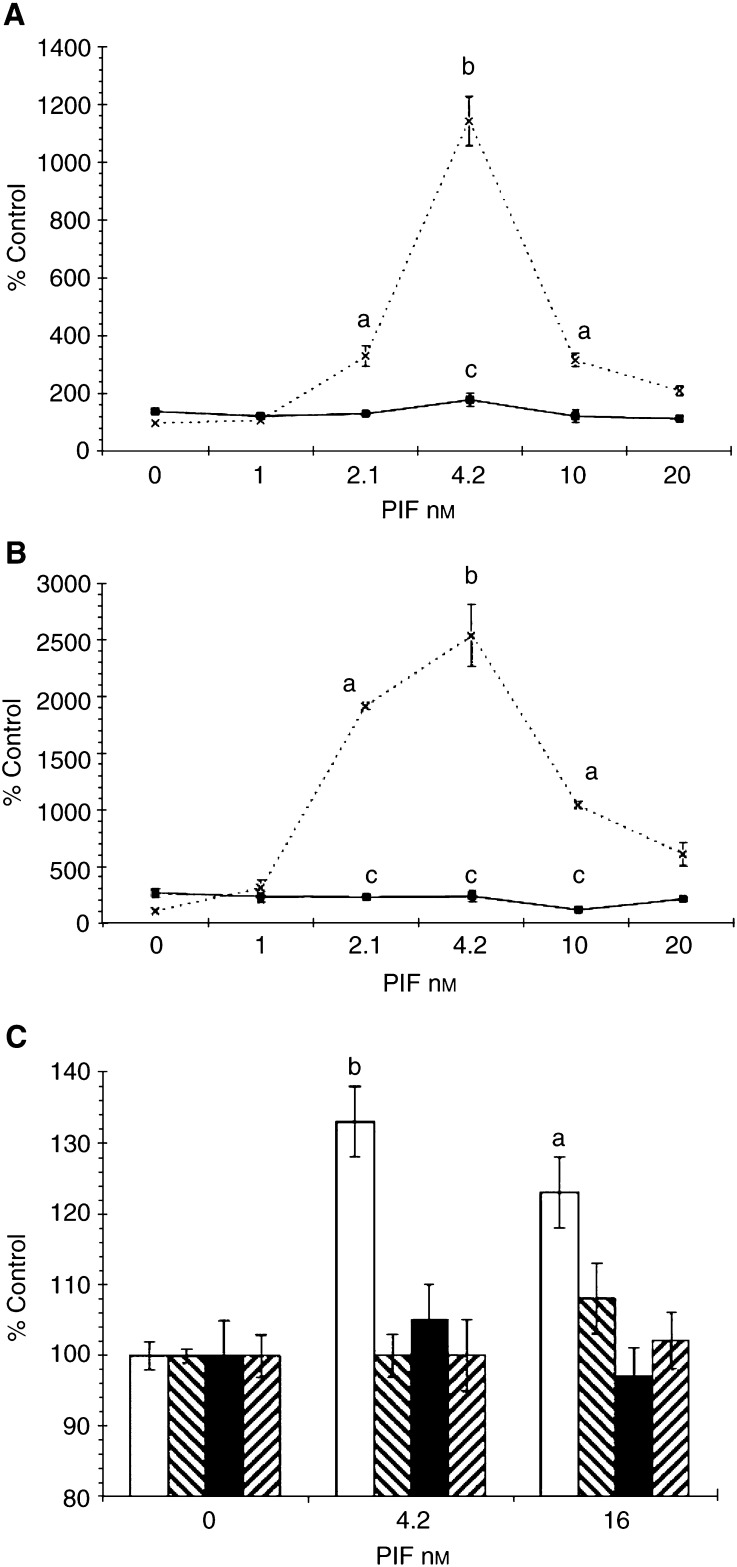
Effect of PIF concentration on the chymotrypsin-like enzyme activity of the proteasome in C_2_C_12_ myotubes in the absence (×) and presence (▪) of (**A**) quinacrine (5 *μ*M) or (**B**) trifluoroacetyl AA (20 *μ*M). (**C**) The effect of quinacrine (5 *μ*M) (□), trifluoroacety l AA (20 *μ*M) (▪) and PD98059 (10 *μ*M) (□) on protein degradation determined by the release of phenylalanine as previously described (Gomes-Marcondes *et al*, 2002) in murine myotubes in the presence of PIF. The experiments were repeated three times (*n*=9). Differences from control are indicated as a, *P*<0.01 and b, *P*<0.001, while differences from those in the absence of the inhibitors are indicated as c, *P*<0.0001. The inhibitors were added to the cells 2 h prior to PIF.

The effect of quinacrine, a nonspecific PLA_2_ inhibitor on proteasome functional activity (‘chymotrypsin-like’ enzyme activity), in the presence of PIF is shown in Figure 1A

**Figure 2:**
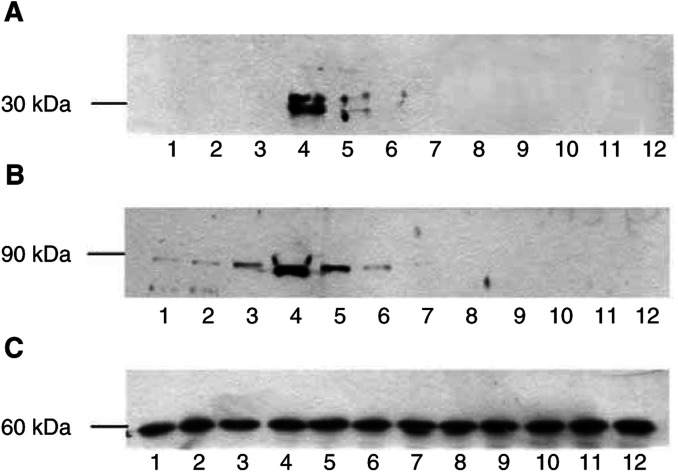
Western blot of soluble extracts of C_2_C_12_ myotubes treated with 0 (lanes 1 and 7); 1.0 (lanes 2 and 8); 2.1 (lanes 3 and 9); 4.2 (lanes 4 and 10); 10 (lanes 5 and 11) or 20 nM PIF (lanes 6 and 12) in the absence (lanes 1–6) or after 2 h pretreatment with trifluoroacetyl AA (20 *μ*M) (lanes 7–12). Bands were detected using either antibody to 20S proteasome *α*-subunits (**A**), iPLA_2_ (**B**) or *β*-tubulin (**C**). The blots shown are representative of at least three separate experiments.

. As previously reported (Gomes-Marcondes *et al*, 2002), PIF produced a significant increase in proteasome activity at concentrations between 2 and 10 nM, with a peak of activity at 4 nM, while both higher and lower concentrations had no effect. At a concentration of 5 *μ*M, quinacrine completely attenuated the increase in ‘chymotrypsin-like’ enzyme activity in the presence of PIF (Figure 1A), as did the cytosolic PLA_2_ inhibitor trifluroacetyl AA (Figure 1B). This might be expected, since cytosolic PLA_2_ exhibits a high selectivity towards the cleavage of unsaturated fatty acids and in particular AA (Glasser *et al*, 1993). Both quinacrine and trifluroacetyl AA also attenuated the PIF-induced increase in protein degradation (Figure 1C). Trifluoroacetyl AA also attenuated the increase in 20S proteasome expression in the presence of PIF (Figure 2A

) and the increase in calcium-independent cytosolic PLA_2_ (iPLA_2_) (Figure 2B) as detected by Western blotting.

**Figure 3:**
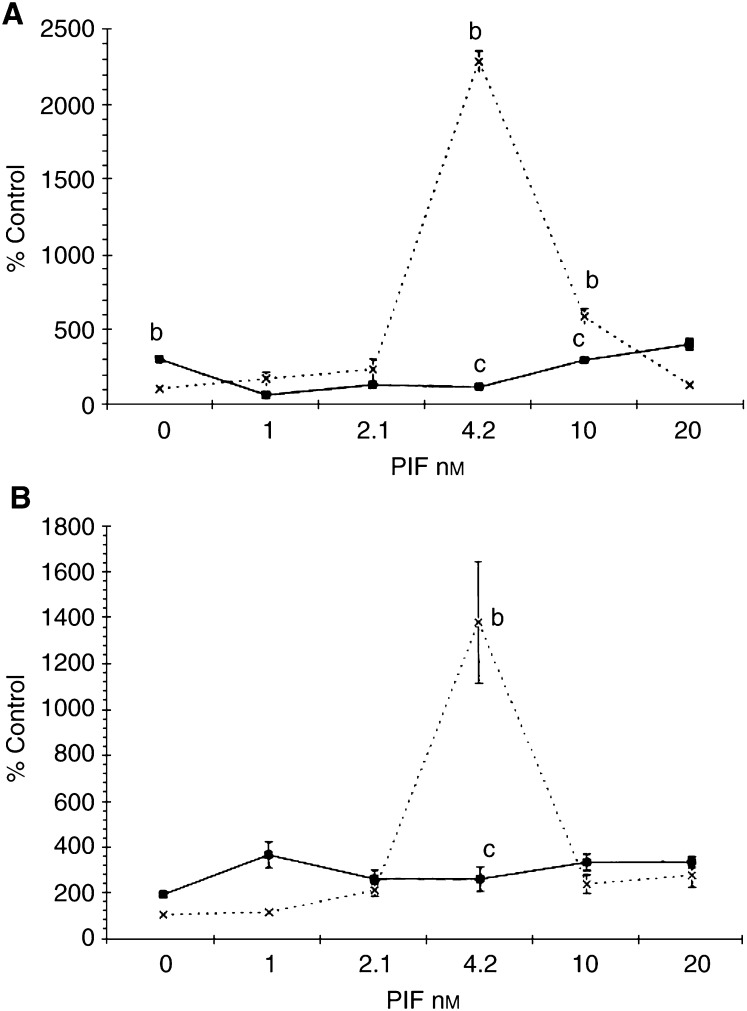
Effect of PIF concentration on the chymotrypsin-like enzyme activity of the proteasome in C_2_C_12_ myotubes in the absence (×) or presence (▪) of U7311 (5 *μ*M) (**A**) or D609 (200 *μ*M) (**B**). The inhibitors were added to the cells 2 h prior to PIF. The experiment was repeated three times (*n*=9). Differences from the control are indicated as b, *P*<0.001, while differences from those in the absence of inhibitors are indicated as c, *P*<0.001.

The PIF-induced increase in ‘chymotrypsin-like’ enzyme activity was also inhibited by U-73122 (Figure 3A

), which inhibits agonist-induced PLC activation (Yule and Williams, 1992), and D609 (Figure 3B), a selective inhibitor of phosphatidylcholine (PC)-specific PLC (Sauer *et al*, 1984). These results suggest that both PLA_2_ and PLC are involved in the hydrolysis of AA from membranes of muscle cells in response to PIF, and that this is important in proteasome expression.

**Figure 4:**
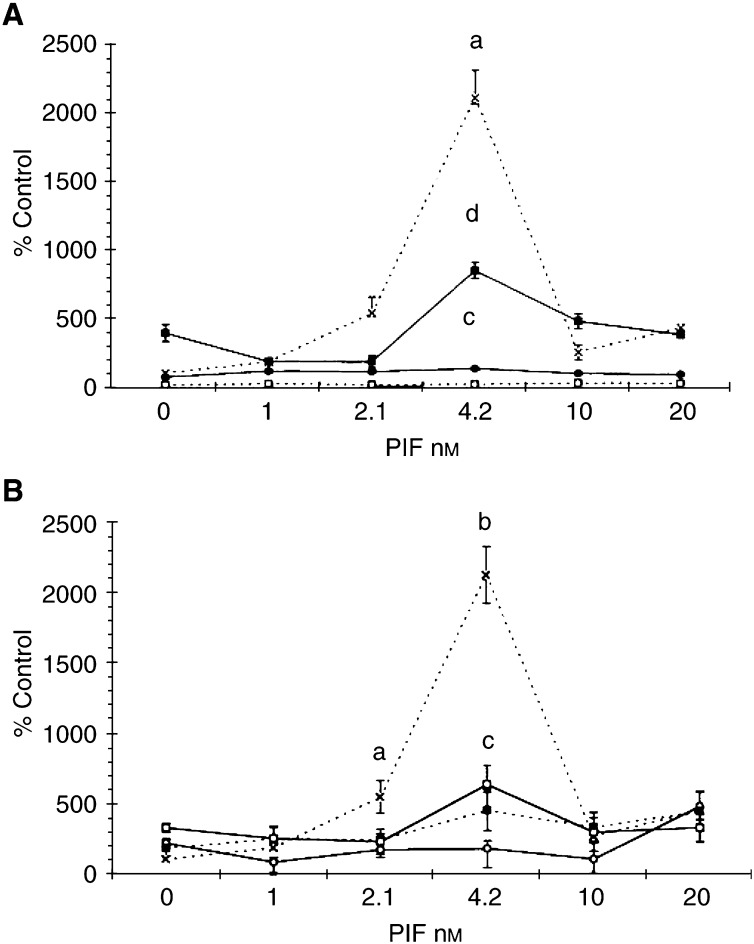
Effect of the concentration of PIF on the chymotrypsin-like enzyme activity in C_2_C_12_ myotubes in the absence (×) or presence of genistein 30 (▪), 100 (•) or 300 (▭)*μ*M (**A**) or 30 (□), 100 (▪) or 300 (○)*μ*M tryphostin A23 (**B**), where *n*=9. Differences from the control are indicated as a, *P*<0.05 and b, *P*<0.005, while differences in the presence of the inhibitor are indicated as c, *P*<0.005 or d, *P*<0.05.

If PLC is involved in PIF-induced proteasome induction, this suggests that PKC may also be required for intracellular signal transduction. We have previously shown (unpublished results) that PKC is involved in PIF-induced proteasome expression and therefore the effect of two tyrosine-kinase inhibitors genistein and tryphostin A23 on PIF-induced ‘chymotrypsin-like’ enzyme activity was determined (Figure 4

). Both genistein at a concentration of 100 and 300 *μ*M (Figure 4A) and tryphostin A23, also at a concentration greater than 100 *μ*M (Figure 4B), completely attenuated the PIF-induced increase in proteasome activity. These results suggest that protein tyrosine kinase is also involved in PIF-induced proteasome expression.

**Figure 5:**
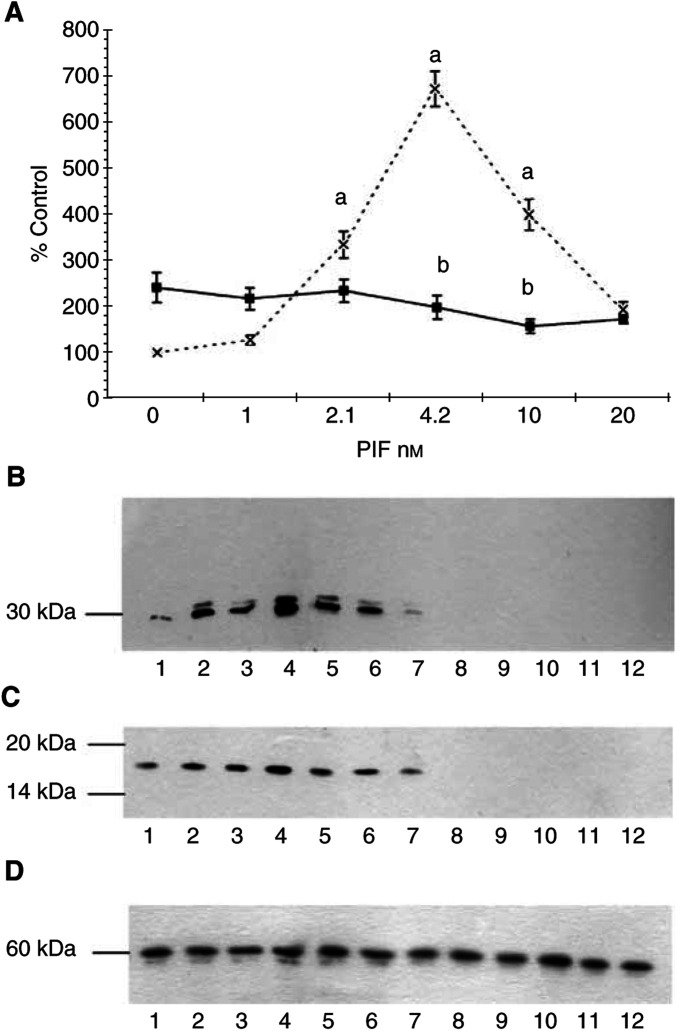
(**A**) Effect of PIF concentration on the chymotrypsin-like enzyme activity of the proteasome in C_2_C_12_ myotubes in the absence (×) or presence (▪) of 10 *μ*M PD98059. The experiment was repeated three times (*n*=9). Differences from the control are indicated as a, *P*<0.001, while differences from those in the absence of inhibitor are indicated as b, *P*<0.001. (**B**–**D**) Western blots of soluble extracts of C_2_C_12_ myotubes treated with 0 (lanes 1 and 7); 1.0 (lanes 2 and 8); 2.1 (lanes 3 and 9); 4.2 (lanes 4 and 10); 10 (lanes 5 and 11) or 20 nM PIF (lanes 6 and 12) without pretreatment (lanes 1–6) and after pretreatment for 2 h in the presence of 10 *μ*M PD98059 (lanes 7–12). Bands were detected using antibody to 20S proteasome *α*-subunits (**B**), E2_14k_(**C**) or *β*-tubulin (**D**).

**Figure 6:**
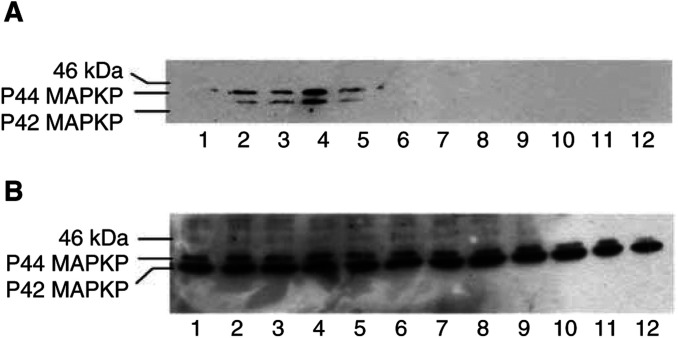
Western blot of active (phosphorylated) ERK1/2 and total ERK1/2 (p44 and p42) (**B**) in soluble extracts of C_2_C_12_ myotubes treated with 0 (lanes 1 and 7); 1.0 (lanes 2 and 8); 2.1 (lanes 3 and 9); 4.2 (lanes 4 and 10); 10 (lanes 5 and 11) or 20 nM PIF (lanes 6 and 12), without pretreatment (lanes (1–6) or after pretreatment for 2 h in the presence of 10 *μ*M PD98059 (lanes 7–12).

Activation of PKC has been shown to activate the ERK and subsequently MAPK (Toker, 1998). To investigate a role for MAPK in PIF-induced proteasome expression, the effect of the selective and cell-permeable inhibitor of MAP kinase kinase (MEK) PD98059 (Kültz *et al*, 1998) was investigated. PD98059 attenuated the PIF-induced increase in ‘chymotrypsin-like’ enzyme activity (Figure 5A

), 20S *α*-subunit expression (Figure 5B) and E2_14k_ (Figure 5C). As shown in Figure 6A

, PIF induced phosphorylation of p44/42 MAPK, while the total MAPK remained unchanged (Figure 6B). The concentration of PIF inducing a maximum phosphorylation of p44/42 MAPK (Figure 6A) (4.2 nM) was the same as that inducing proteasome expression and E2_14k_ (Figure 5). PD98059 completely blocked PIF-induced p44/42 MAPK activation (Figure 6A) as well as proteasome expression (Figure 5), confirming a role for MAPK activation in PIF-induced proteasome expression.

## DISCUSSION

Using murine myoblasts as a surrogate model of skeletal muscle, the induction of protein degradation by PIF was positively correlated with AA release and subsequent conversion to 15(*S*)-HETE (Smith *et al*, 1999). This process was blocked by eicosapentaenoic acid, which also attenuated protein degradation by PIF, both *in vitro* (Smith *et al*, 1999) and *in vivo* (Hussey and Tisdale, 1999). These observations suggest that the formation of eicosanoids from AA was important in PIF-induced protein catabolism, mediated through the upregulation of the ubiquitin–proteasome proteolytic pathway (Lorite *et al*, 2001). Despite the importance of the ubiquitin–proteasome pathway, very little is known about the intracellular signal transduction pathways involved in gene expression. Although glucocorticoids are know to activate the pathway by opposing the suppression of the transcription of proteasome *α*-subunits by nuclear factor-*κ*B (NF-*κ*B) (Du *et al*, 2000) other factors may be involved, since chronic excessive glucocorticoid production, as occurs in Cushing's syndrome, does not increase proteasome expression (Ralliere *et al*, 1997).

**Figure 7:**
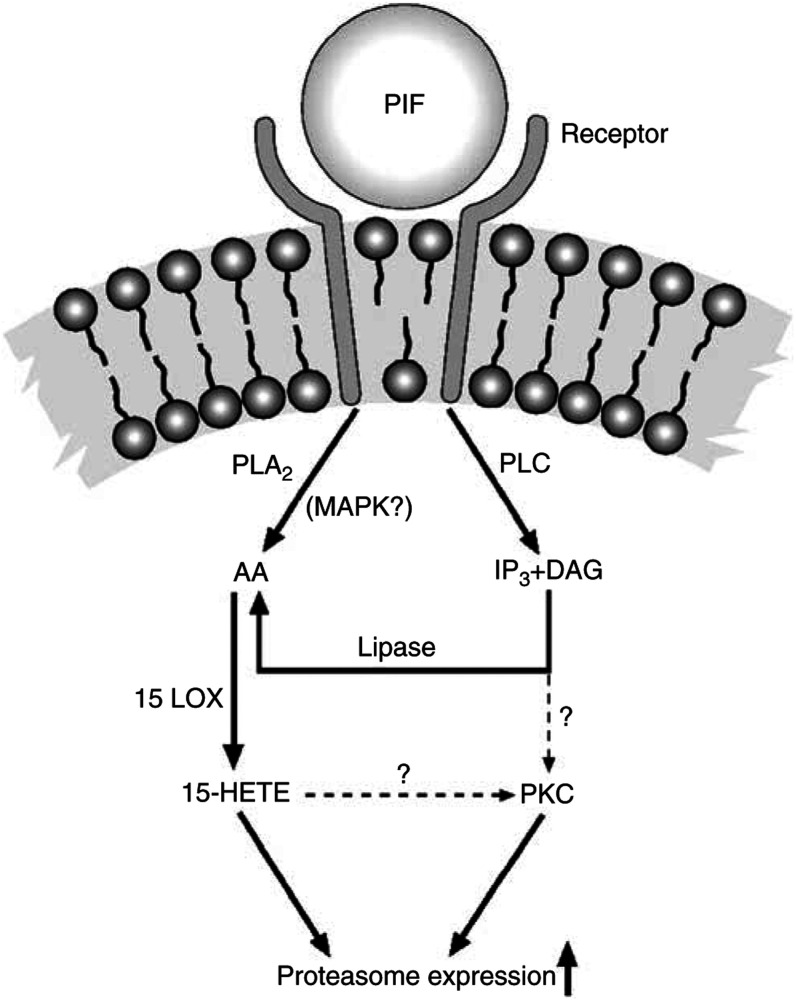
Signal transduction pathways involved in PIF activation of proteasome expression. 15-LOX, 15-lipoxygenase; IP3, inositol 1, 4, 5-trisphosphate.

Arachidonic acid is released from cell membranes by the action of phospholipases. PLA_2_ catalyses the release of fatty acid from the sn-2 position of all membrane phospholipids with the formation of lysophospholipids, while PLC hydrolyses the glycerophosphate ester bond of a variety of phospholipids with the formation of DAG and a phosphate monoester, which can be hydrolysed to AA by DAG lipase (Figure 7

). Quinacrine, a nonspecific inhibitor of PLA_2_, was shown to attenuate PIF-induced proteasome activity, determined by the chymotrypsin-like enzyme activity, and protein degradation, suggesting a role for PLA_2_ in this process. Proteolysis-inducing factor was shown to increase the expression of iPLA_2_ at the same concentrations as those inducing the maximal expression of 20S proteasome *α*-subunits and protein degradation. The induction of iPLA_2_, proteasome expression and protein degradation by PIF were completely inhibited by trifluroacetyl arachidonic acid, an inhibitor of PLA_2_. This suggests that either this derivative of AA is directly downregulating the expression of iPLA_2_ or that AA itself is responsible for stimulating the expression of iPLA_2_. These results confirm that iPLA_2_ is involved in PIF-induced proteasome expression and protein degradation through the release of AA from membrane phospholipids. The activation of PLA_2_ has been shown to involve MAPK (Lin *et al*, 1993), suggesting a relationship between the observed activation of MAPK and PLA_2_ activation by PIF.

In addition to PLA_2_, PLC was also shown to be involved in PIF-induced proteasome expression, as shown by the attenuation of the effect using the PLC inhibitors U73122 and D609, confirming the importance of the release of AA to the overall process. D609 is a selective inhibitor of PC-specific PLC (Sauer *et al*, 1984) and DAG derived from PC-PLC is suggested to provide a positive feedback signal to PKC (Fallman *et al*, 1992), which does not appear to cause downregulation of the enzyme (Daiz-Laviada *et al*, 1990). TNF-*α* induction of ICAM-1 expression in A549 cells involves the activation of PC-PLC, which induces activation of PKC*α* and protein tyrosine kinase (Chen *et al*, 2001). This suggests that the activation of PLC provides a signal for PKC activation, as well as another source of AA. We have recently shown PKC to be involved in PIF-induced proteasome expression (unpublished results), possibly acting as a signal for NF-*κ*B activation (Vertegaal *et al*, 2000). TNF-*α*-induced activation of NF-*κ*B was inhibited by selective inhibitors of cytosolic PLA_2_ (Thommesen *et al*, 1998), suggesting that this pathway may also be involved in NF-*κ*B-activated gene expression. In addition, PC-PLC has been shown to activate protein tyrosine kinase (Chen *et al*, 2001) and ERK (Toker, 1998). Both the tyrosine kinase inhibitors genistein and tryphostin A23 attenuated PIF-induced proteasome expression, suggesting a role for protein tyrosine kinase in this process.

In mammalian cells, three parallel MAPK pathways have been identified, which includes ERKs, p44 MAPK (ERK1) and p42 MAPK (ERK2), stress-activated protein kinase, c-Jun-NH_2_-terminal kinases and the p38 MAPK (Chang and Karin, 2001). Extracellular signal-regulated kinases are activated by growth factors acting via MAPK kinase kinase, (such as Raf) and MEKs are involved in both cell proliferation and differentiation (Chang and Karin, 2001). The pathway has been classically viewed to respond to growth factors with the activation of tyrosine kinase receptors acting through small G proteins, such as Ras, leading to the activation of Raf, which then phosphorylates and activates MEK1 and MEK2, which in turn phosphorylate and activate ERK1 and ERK2. The present study shows that PIF induces phosphorylation of ERK1 and ERK2 at the same concentrations as those inducing proteasome expression and that PD98059, a selective inhibitor of MEK (Kültz *et al*, 1998), attenuated both the PIF-induced activation or ERK1 and ERK2, and the induction of proteasome expression. This suggests that PIF induces proteasome expression through the MAPK pathway. The mechanism by which this occurs is not known, but the MAPK/ERK pathway has been classically viewed to respond to growth factors with the activation of tyrosine kinase receptors acting through small G proteins, such as Ras (Chang and Karin, 2001). The involvement of tyrosine kinase in PIF induction of proteasome expression suggests the operation of a similar pathway. These results provide some information on the intracellular signalling pathways involved in the induction of proteasome expression by PIF (Figure 7).

PIF has been shown to bind to a membrane receptor on skeletal muscle (unpublished observations), although the nature of this receptor and the relationship to PLA_2_ aret known. Although we have only been able to demonstrate PIF production by cachexia-inducing tumours (Cariuk *et al*, 1997), it may be important during embryonic development. Proteolysis-inducing factor has been shown to be expressed during the embryonic period E8–E9 in mice, peaking during E8.5, a crucial stage in the patterning and eventual development of skeletal muscle (Watchorn *et al*, 2001). It seems that receptors for PIF required at this stage are still expressed in adult skeletal muscle even in the absence of the agonist. Although PIF production ceases in the adult, the peptide chain, which is devoid of proteolytic activity (Todorov *et al*, 1996a), is still synthesised as the antimicrobial peptide dermicidin (Schittek *et al*, 2001) or as Y-P30, a neuronal survival peptide (Cunningham *et al*, 2002). The acquisition by certain tumours of the enzymes necessary to glycosylate this peptide chain leads to PIF expression and breakdown of skeletal muscle.
